# DiabetesWise: An innovative approach to promoting diabetes device awareness

**DOI:** 10.1111/1753-0407.13401

**Published:** 2023-05-04

**Authors:** Jessie J. Wong, Ananta Addala, Sarah J. Hanes, Sara Krugman, Diana Naranjo, Sierra Nelmes, Kyle Jacques Rose, Molly L. Tanenbaum, Korey K. Hood

**Affiliations:** ^1^ Department of Pediatrics, Division of Endocrinology and Diabetes Stanford University School of Medicine Stanford California USA; ^2^ Healthmade Design Oakland California USA; ^3^ EiR Visiting Faculty, INSEAD Healthcare Management Fontainebleau France; ^4^ Stanford Diabetes Research Center Stanford University School of Medicine Stanford California USA; ^5^ Department of Medicine, Division of Endocrinology, Gerontology, and Metabolism Stanford University School of Medicine Stanford California USA

**Keywords:** continuous glucose monitor (CGM), diabetes distress, insulin, insulin pump, type 1 diabetes, type 2 diabetes, 动态血糖监测, 糖尿病困扰, 胰岛素, 胰岛素泵, 1型糖尿病, 2型糖尿病

## Abstract

**Background:**

DiabetesWise is an unbranded, data‐driven online resource that tailors device recommendations based on preferences and priorities of people with insulin‐requiring diabetes. The objective of this study is to examine whether DiabetesWise increases uptake of diabetes devices, which are empirically supported to improve glycemic and psychosocial outcomes.

**Methods:**

The sample included 458 participants (M_age_ = 37.1, SD = 9.73; 66% female; 81% type 1 diabetes) with insulin‐requiring diabetes and minimal diabetes device use at enrollment. Participants used DiabetesWise and completed online surveys. Chi‐square and *t* tests evaluated requests for a device prescription, receiving a prescription, and starting a new device at 1 and 3 months post use. Baseline predictors of these variables and past use of continuous glucose monitors (CGMs) and changes in diabetes distress post use were also examined.

**Results:**

Within the first month of interacting with DiabetesWise 19% of participants asked for a prescription for a diabetes device. This rate rose to 31% in the first 3 months. These requests resulted in 16% of the sample starting a new device within the first 3 months. Whereas several factors were associated with prior CGM use, receiving a prescription, and starting a new device, more diabetes distress (*t*(343) = −3.13, *p* = .002) was the only factor associated with asking for a prescription. Diabetes distress decreased after interacting with DiabetesWise within 1 month (*t*(193) = 3.51, *p* < .001) and 3 months (*t*(180) = 5.23, *p* < .001).

**Conclusions:**

Within 3 months of interacting with DiabetesWise, one in three participants had requested a prescription for a new diabetes device and average distress levels were reduced, indicating benefit from this low‐intensity online platform.

## Graphical abstract

**Highlights**

- About a third of participants requested diabetes device prescriptions with 3 months of being invited to interact with DiabetesWise.
- Many factors predicted prior CGM use but diabetes distress was the only predictor of requesting a prescription after interacting with DiabetesWise.
- Diabetes distress decreased over time after interacting with DiabetesWise.

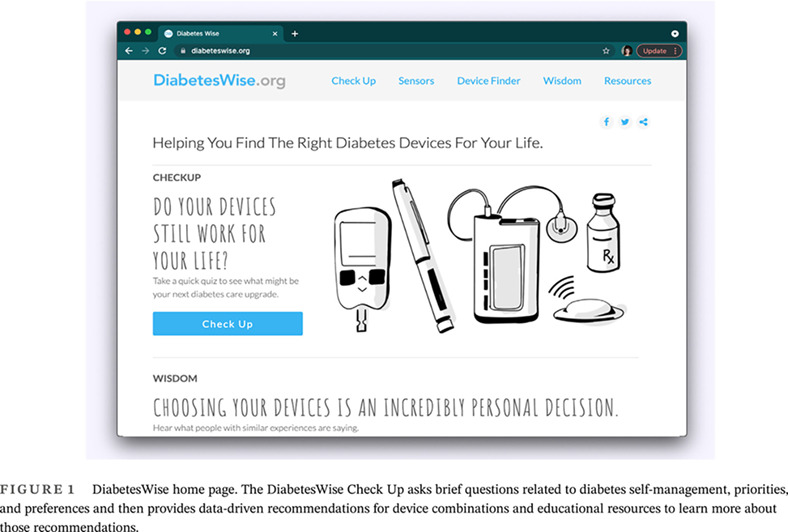

## 摘要

背景:DiabetesWise是一个无品牌的, 以数据驱动的在线资源, 根据胰岛素需求者的偏好和优先事项, 量身定制设备推荐。本研究的目的是研究DiabetesWise是否能增加胰岛素需求者使用胰岛素治疗设备的比率, 这些设备经过实证支持可改善血糖和心理社会结果。

方法:样本包括458名参与者(平均年龄=37.1岁, 标准差=9.73；66%女性；81%为1型糖尿病患者), 这些参与者在入组时使用胰岛素治疗且对糖尿病设备的使用较少。参与者使用DiabetesWise并完成在线调查。使用卡方检验和t检验评估使用DiabetesWise后1个月和3个月内器械处方请求, 接受处方和开始使用新设备的比率。还研究了这些变量的基线预测因素, 过去连续血糖监测(CGM)使用情况以及使用后糖尿病困扰变化。

结果:在与DiabetesWise互动的第一个月内, 有19%的参与者要求处方以获取糖尿病设备。在前三个月内, 这一比率上升至31%。这些要求导致了样本中16%的参与者在前三个月内开始使用新的设备。虽然几个因素与先前CGM使用, 接受处方和开始使用新设备有关, 但更多的糖尿病困扰(t(343)=‐3.13, p=0.002)是要求处方的唯一相关因素。与DiabetesWise互动后, 一个月内(t(193)=3.51, p<0.001)和三个月内(t(180)=5.23, p<0.001), 糖尿病困扰程度减少。

结论:在与DiabetesWise互动的三个月内, 三分之一的参与者要求处方以获取新的糖尿病设备, 并且平均困扰程度降低, 表明这个低强度在线平台具有益处。

## 1 INTRODUCTION

Diabetes technologies provide a myriad of benefits to people living with diabetes.^1^, ^2^, ^3^, ^4^ Continuous glucose monitors (CGMs) can reduce glycated hemoglobin (HbA1c) and hypoglycemia and increase the percentage of time‐in‐range for people living with type 1 and type 2 diabetes, as well as increase diabetes‐related treatment satisfaction and quality of life.^5^ Studies of insulin pumps^6^ and smart systems^7^, ^8^ show similar glycemic and psychosocial benefits. Unfortunately, uptake of these technologies has been limited and access has been inequitable. Cost, insurance coverage, and provider attitudes^9^ as well as patient characteristics including attitudes toward diabetes technology and younger age^10^ have served as barriers to technology uptake for many. Diabetes device education and support for attaining a device has traditionally fallen to diabetes care providers who may have limited time and resources and may not be informed about the latest devices available. Diabetes device companies often promote awareness and education for their respective devices, but financial interests can narrow and bias the scope of the information they provide. As such, many people living with diabetes remain unaware of their device options and unable to benefit from advancements in these technologies.

**FIGURE 1:**
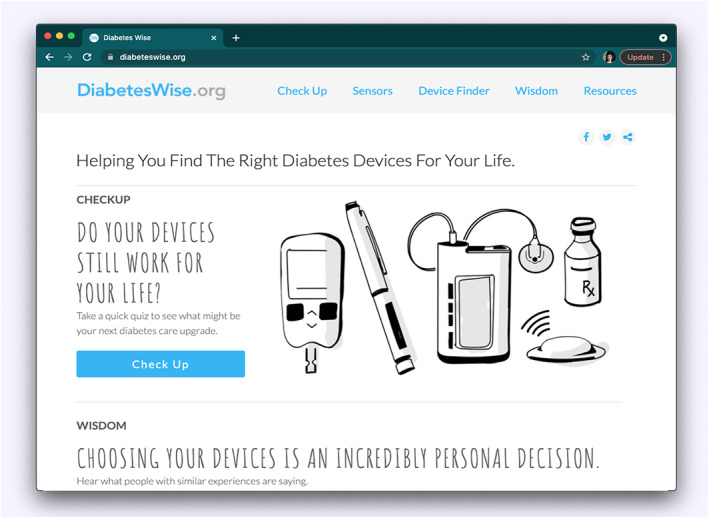
DiabetesWise home page. The DiabetesWise Check Up asks brief questions related to diabetes self‐management, priorities, and preferences and then provides data‐driven recommendations for device combinations and educational resources to learn more about those recommendations.

**FIGURE 2:**
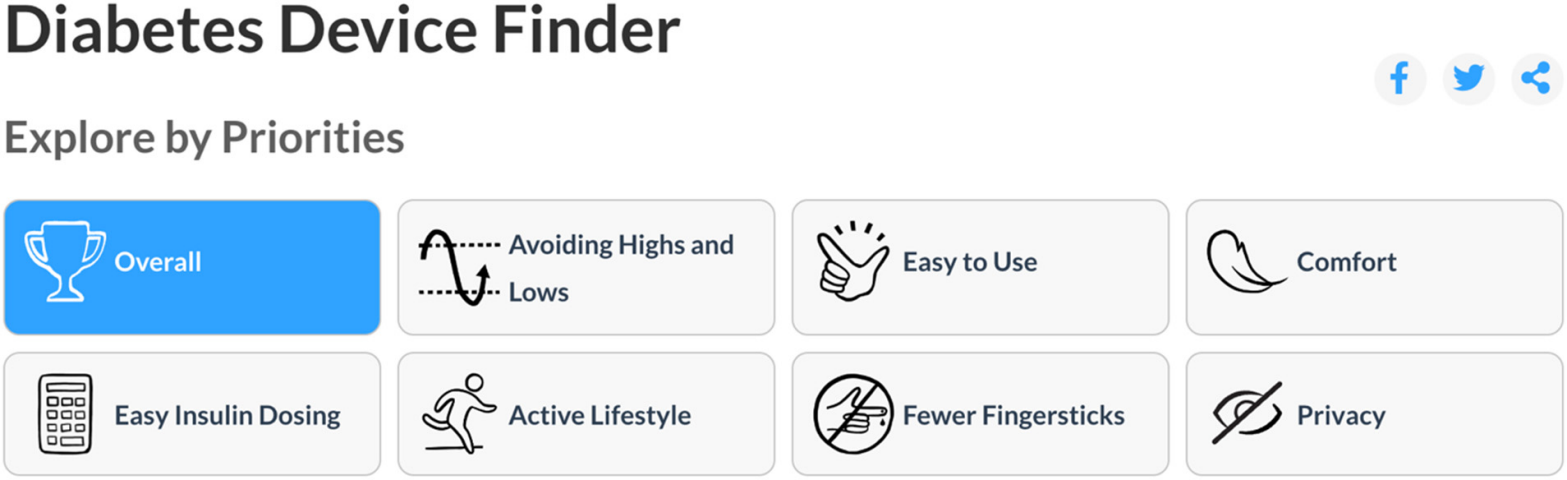
DiabetesWise's diabetes device finder. The Diabetes Device Finder allows users to browse and search for diabetes devices based on specific priorities. It also includes filters to select specific brands and/or devices.

**FIGURE 3:**
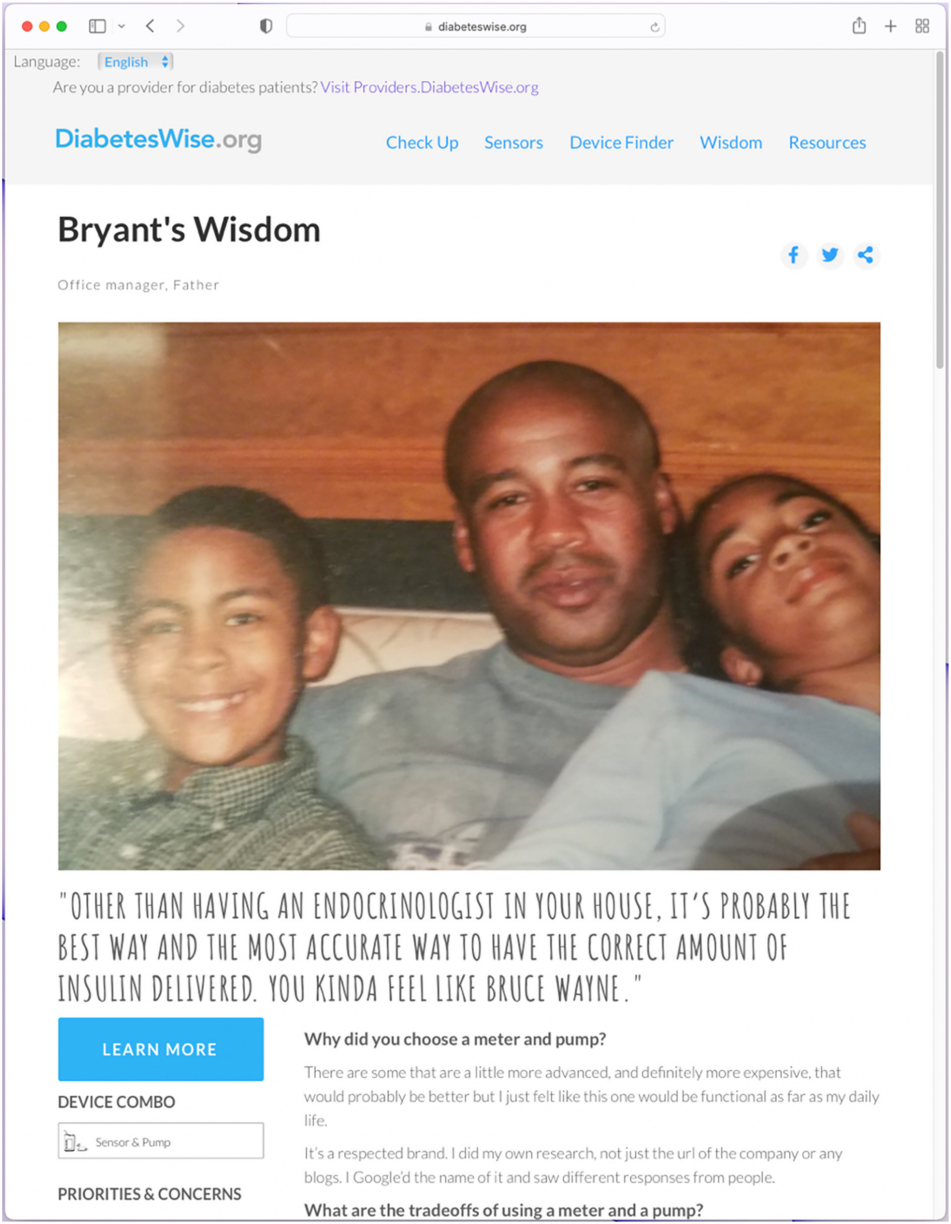
Example of a personal story and account of diabetes management experience (“wisdom”) provided by DiabetesWise. DiabetesWise “wisdom” shares personal accounts of people's experiences with diabetes management, including personal preferences and lived experiences. These accounts highlight the varying and personalize nature of selecting devices and device combinations.

DiabetesWise is an online resource designed specifically to address and overcome barriers to device uptake (Figure 1). It was created with funding from the Helmsley Charitable Trust and is not connected to any device manufacturer. As such, it is a free, unbranded resource for people with insulin‐requiring diabetes. Data derived from multiple studies^10^, ^11^, ^12^ and user experiences^13^, ^14^ informed the underlying algorithm to match preferences with devices by considering critical factors such as diabetes distress.^13^ This algorithm was used to make data‐driven recommendations based on user responses to the brief survey or “Check Up” on the platform. The Check Up asked questions about diabetes management, preferences, and needs, especially related to device use and nonuse. Educational content is another primary component of the platform, including the advantages and disadvantages of specific devices as well as every possible device combination. The “Diabetes Device Finder” helps user browse this content based on users' selected priorities and allows them to filter based on specific brands/types/models of devices (Figure 2). Another key component of DiabetesWise includes personal stories and accounts (“wisdom”) of diabetes management experiences with various devices (Figure 3). These stories serve to emphasize the personalized nature of diabetes management, reflect variability in diabetes management preferences, and empower users to identify and prioritize their personal needs and goals. Overall, DiabetesWise seeks to empower people with diabetes to understand their options and make informed decisions about their self‐management choices, regardless of whether those decisions include device adoption or not.

The current study sought to evaluate whether DiabetesWise promotes steps toward diabetes technology uptake among its users. To this end, we examined (1) whether interacting with DiabetesWise led to action toward device uptake, (2) factors associated with device uptake after interacting with DiabetesWise, and (3) changes in diabetes distress following engagement with DiabetesWise. By understanding these factors and processes, we hope to evaluate the potential effects of interacting with DiabetesWise and expand our empirical understanding of personal and contextual barriers and facilitators to diabetes device uptake.

## 2 METHODS

Potential participants were recruited through online postings on social media. Inclusion criteria were at least 18 years of age, diagnosis of type 1 or type 2 diabetes, using insulin, and US resident. Exclusion criteria were current consistent CGM use (at least 5 days per week) and/or current closed loop or automated insulin delivery system use at the time of enrollment. If eligible, participants were directed to online documents to review and provide consent prior to study enrollment. Once enrolled, participants completed an online baseline survey, completed at‐home blood collection to measure HbA1c, and received instructions to access DiabetesWise. For this study, the public facing DiabetesWise website was cloned and study access required an initial login and password, which were provided by the study coordinator. Only study participants could access the cloned website, which permitted tracking of activity on the website. Follow‐up surveys were administered monthly for 3 months. As previously described, the platform provided a Check Up (Figure 1) with data‐driven recommendations, ability to find and compare educational content, through the Diabetes Device Finder (Figure 2), as well as personal accounts related to diabetes device use (Figure 3). All study activities were conducted remotely, allowing for a geographically diverse sample and minimal participant burden. The study protocol was approved by the Stanford University Institutional Review Board (#52851).

### 2.1 Measures

Demographic measures of gender, age, race/ethnicity, education, employment, income, insurance type, diabetes type, provider type, and medical setting were all captured via self‐report survey at baseline.

Participants also self‐reported whether (1) they asked a provider for a prescription for a diabetes device/system; (2) they received a new prescription for a diabetes device/system from their provider, and (3) they started using a new diabetes device/system. They also reported on the type of new device/system (CGM, insulin pump, and/or smart system) they had started using, if any. These variables were captured through surveys administered monthly after interacting with DiabetesWise.

Diabetes distress was measured by the 28‐item Type 1 Diabetes Distress Scale (T1‐DDS),^15^ a validated measure that captures the emotional distress and burden of managing type 1 diabetes.^16^ Given that the current study included only participants with type 2 diabetes who had insulin‐requiring type 2 diabetes, we decided that the T1D‐DDS would be an appropriate questionnaire for the entire sample. This scale provides a continuous score of distress severity, which can be converted into a binary measure based on the clinical cutoff of ≥2. This scale was included in the survey administered at baseline and monthly follow‐ups. Cronbach's alpha for this sample was 0.95 at baseline as well as 1 and 3 months post use, demonstrating strong internal consistency.

### 2.2 Analytic approach

Chi‐square and *t* tests evaluated categorical and continuous factors, respectively associated with past CGM use and requests for and receipts of prescriptions as well as new device uptake. Paired *t* tests were used to compare diabetes distress at follow‐ups with baseline levels of distress. All analyses were conducted in SPSS version 27 using pairwise deletion to handle missing data. Power analyses revealed that the current sample was statistically powered to detect even a small (*d* = 0.33) effect size.

## 3 RESULTS

The current sample included 458 participants (Table 1). All participants required insulin and most had a diagnosis of type 1 diabetes (81%). The majority of participants self‐identified as female (66%) and non‐Hispanic White (82%). Participant ages ranged from 18 to 69 with an average age of 37 years. Participants were mostly employed (72%) with private insurance (78%). Most participants received diabetes care from a specialty diabetes practice (62%) although a large number received diabetes care from their primary care provider (48%). Most diabetes care was provided in private and community health centers (59%) rather than academic settings or large medical centers.

**TABLE 1:** Baseline sample characteristics

Baseline characteristic	Full sample *N* = 458			Type 1 subsample *n* = 370			Type 2 subsample *n* = 88		
	Mean (SD)/%	Min	Max	Mean (SD)/%	Min	Max	Mean (SD)/%	Min	Max
Type 1 diabetes	80.8%			100%			0%		
Gender									
Female	66.4%			64.3%			75.0%		
Male	32.3%			34.6%			22.7%		
Nonbinary	1.1%			0.8%			2.3%		
Prefer not to disclose (*n* = 1)	0.2%			0.3%			0%		
Age (years)	37.1 (9.74)	18.2	69.3	35.5 (9.26)	18.2	65.7	43.9 (8.78)	24.2	69.3
Race or ethnicity; *n* = 455									
White, non‐Hispanic	81.8%			84.2%			71.6%		
Hispanic	5.9%			5.2%			9.1%		
Multiracial	4.4%			4.4%			4.5%		
Asian/Pacific Islander	4.0%			3.3%			6.8%		
Black, non‐Hispanic	3.1%			2.5%			5.7%		
Other (*n* = 1)	0.2%			0%			1.1%		
Education									
Some college or less	33.6%			34.6%			29.5%		
Trade/associate/bachelor's	49.2%			49.2%			48.9%		
Graduate degree	17.2%			16.2%			21.6%		
Employed	72.1%			71.4%			75.0%		
Income (>$100 k); *n* = 434	19.6%			20.3%			16.7%		
Insurance (private)	77.7%			77.6%			78.4%		
HbA1c; *n* = 262	7.77 (1.76)	4.1	14.7	7.65 (1.62)	4.1	12.5	8.26 (2.19)	4.7	14.7
Diabetes distress scale	2.91 (1.05)	1.0	6.0	2.78 (1.05)	1.0	6.0	2.92 (1.07)	1.0	5.5
Diabetes distress cutoff	74.2%			73.5%			77.3%		
Endocrinology provider (vs primary care)	62.4%			67.8%			39.8%		
Medical setting	41.3%			43.5%			31.8%		

*Note*: Medical setting = academic/large medical center vs private practice/community health center.

### 3.1 Prior CGM use

Less than 2% of the sample was using CGM (< 5 days per week) at the start of the study and 39% had a history of CGM use. People who reported prior CGM use were more likely to be female (73% vs 62%, χ ^2^ (1)=6.03, *p* = .014), younger (35.1 vs 38.5, *t*(456), *p* < .001), have type 1 diabetes (92% vs 73%, χ ^2^ (1)=23.54, *p* < .001), and receive care in a large/academic medical center (79% vs 51%, χ ^2^ (1)=36.03, *p* < .001) compared to people who reported no prior CGM use.

### 3.2 Requesting prescriptions

**FIGURE 4:**
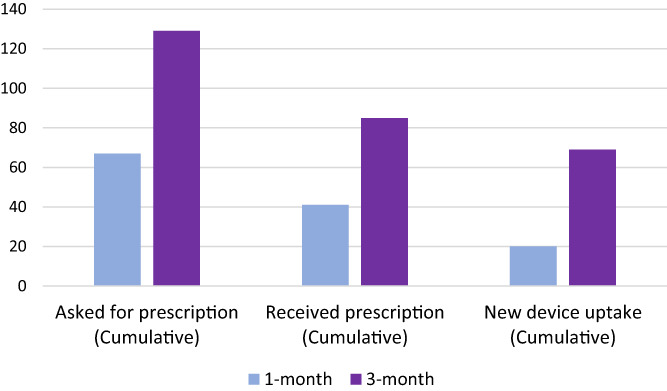
Number of prescription requests and receipt and new device uptake at 1 and 3 months.

Within the first month after interacting with DiabetesWise, 67 (19%) participants asked their diabetes care provider for a prescription for a new diabetes device (Figure 4). Within the first 3 months following DiabetesWise engagement, 31% of all participants asked for a prescription for a new diabetes device. Further details are reported in Table 2.

**TABLE 2:** Prescription requests and receipt and new device uptake at 1 and 3 months.

One month (*n* = 388)			Three months (*n* = 420)		
Asked for prescription (*n* = 345)	67	19%	Asked for prescription	129	31%
Received prescription(s)	41	11%	Received prescription(s)	85	20%
Type			Type		
CGM	36	88%	CGM	30	81%
Insulin pump	13	32%	Insulin pump	17	46%
Smart system	1	2%	Smart system	1	5%
Started new device(s)	20	5%	Started new device(s)	69	16%
Type			Type		
CGM	20	100%	CGM	64	93%
Insulin pump	1	5%	Insulin pump	10	15%
Smart system	0	0%	Smart system	2	3%

Abbreviation: CGM, continuous glucose monitor.

### 3.3 Receiving prescriptions

A total of 41 participants (11%) reported receiving new prescriptions within the first month. Of those, 88% of the prescriptions were for CGM. An additional 32% requested prescriptions for insulin pumps and 2% for smart systems.

A cumulative 85 participants (20%) received a new prescription across the 3‐month study period and 81% of those prescriptions were for CGM. Another 46% received prescriptions for an insulin pump and 3% for smart systems.

### 3.4 New device uptake

Twenty (5%) participants began new devices in the first month. All of these participants who started a new device within the first month started a new CGM and 5% also started a new insulin pump. Device uptake within the first month was significantly associated with younger age. Participants who started a new device had an average age of 32.9 (SD = 10.23) years compared to 37.4 (SD = 9.64) years among those who did not start a new device (t(386) = 2.01, *p* = .045).

Sixty‐nine (16%) participants began new devices across the 3‐month study period. Of these new users, 83% started a new CGM only, 7% started a new CGM and insulin pump, 7% started a new insulin pump only, and 3% started a new CGM and smart system.

### 3.5 Factors associated with requests, prescriptions, and new device uptake

Diabetes distress was the only baseline factor associated with whether participants asked for a new device prescription. Higher baseline diabetes distress was reported among participants who requested a prescription for a diabetes device within the first month (3.11 vs 2.67, *t*(343) = −3.13, *p* = .002) and first 3 months (3.06 vs 2.67, *t*(418) = −3.53, *p* < .001) compared to participants who did not request a prescription following DiabetesWise engagement. In addition, diabetes distress was correlated only with requests, not with receiving a prescription or starting a new device.

Whether a participant received a prescription from a provider varied by diabetes type. Specifically, 23% of participants with type 1 diabetes received a prescription in the first 3 months compared to 11% of participants with type 2 diabetes (χ ^2^ (1)=6.15, *p* = .013).

Medical setting was correlated with receiving a prescription and new device uptake. A total of 90% of the participants who received a prescription for a diabetes device in the first month after interacting with DiabetesWise received care from a specialty clinic, compared to 61% of participants who did not receive a prescription (χ ^2^ (1)=14.0, *p* < .001). In the first 3 months, those who received a prescription (85% vs 56%, χ ^2^ (1)=23.1, *p* < .001) and started a new device (75% vs 60%, χ ^2^ (1)=6.1, *p* = .013) also more commonly received care through a specialty clinic than those who did not receive a prescription or start a new device, respectively.

Insurance type and race or ethnicity were not significantly related to any of these processes.

### 3.6 Diabetes distress

Diabetes distress was found to decrease over time following engagement with DiabetesWise. Reductions in distress severity were observed from baseline to 1‐month (2.82 vs 2.63, *t*(193) = 3.51, *p* < .001) and 3‐month (2.70 vs 2.40, *t*(180) = 5.23, *p* < .001) follow‐ups. Similarly, the proportion of participants who met or exceeded the clinical cutoff (score ≥2) reduced from baseline to 1‐month (74% vs 66%; χ ^2^ (1)=69.08, *p* < .001) and 3‐month (74% vs 61%; χ ^2^ (1)=50.98, *p* < .001) follow‐ups.

## 4 DISCUSSION

Diabetes device uptake has been slow and inequitable. Socioeconomic,^17^ racial, ethnic,^18^, ^19^ gender,^19^ and geographic‐related^19^ disparities in diabetes technology use are well documented. Unfortunately, an uptake process that relies on medical providers, health care systems, and device companies for exposure to and education about diabetes technologies remains highly vulnerable to the biases that perpetuate these disparities. DiabetesWise presents an alternative pathway to promote diabetes technology awareness and education by centering on personal experiences and preferences through an unbranded and user‐friendly platform. In this way, DiabetesWise can democratize diabetes technology knowledge to expand access and uptake. The current study examined whether interacting with DiabetesWise leads to device uptake, factors associated with uptake, and changes in diabetes distress. Results showed that 19% of participants asked for a device prescription in the first month, which rose to 31% in the first 3 months following initial engagement with DiabetesWise. These requests were met with slightly lower rates of receiving a prescription, including 11% in the first month and 20% in the first 3 months. Ultimately, 5% of participants started using a device within the first month and this increased to 16% after 3 months. The majority (83%) of new device users adopted CGM only. Overall, these findings suggest that resources such as DiabetesWise can promote interest and uptake of diabetes devices, particularly CGM.

Regarding past device use, results showed that CGM use prior to interacting with DiabetesWise was associated with being female, younger age, type 1 diabetes, and receiving care in a large/academic medical center. These findings coincide with prior observations of females being more likely to use insulin pumps than males^19^ and greater CGM^20^ and insulin pump use^21^ among younger compared to older people with type 1 diabetes. Higher baseline diabetes distress was the only factor associated with whether participants asked their provider for a new device prescription after interacting with DiabetesWise. Factors related to prescription receipt and device uptake included age, diabetes type, and whether diabetes care was provided in primary care or endocrinology. This pattern contrasts previous observational results in which uptake was related to insurance type and race/ethnicity,^17^, ^20^ suggesting uptake related to DiabetesWise use may vary from uptake following traditional pathways. Together, these findings may also suggest that DiabetesWise can promote users' self‐advocacy, though further changes are needed to ensure equity related to prescription and device receipt.

The finding that diabetes distress was the only factor significantly associated with new prescription requests after interacting with DiabetesWise is quite notable. Whereas previous research has found that greater diabetes distress is associated with less self‐management,^15^, ^22^ the current findings suggest that interacting with DiabetesWise may empower people to actively change their diabetes management, especially when they experience elevated diabetes distress. These people may have a greater need for diabetes devices and/or change to their management regimen and DiabetesWise may provide the resources, support, and encouragement to act on such needs. If so, DiabetesWise seems to function as designed: based on the user's needs and experiences. This potential effect of DiabetesWise is particularly promising given that diabetes technology uptake and continued use prospectively predict reduced diabetes distress over time.^7^, ^24^, ^25^ Our findings that distress was not related to prior CGM use, receiving a prescription, or device uptake are consistent with cross‐sectional observational findings that have also shown diabetes distress levels to be unrelated to diabetes technology use.^13^, ^23^ These overall patterns suggest that diabetes distress may have contrasting and reciprocal influences with diabetes technology needs, uptake, and ongoing use. Providers, insurance, cost, and other powers might influence prior CGM use or receiving a new prescription or device but not whether a participant asks a provider for a prescription. Requesting a prescription was the only variable related to device uptake that is within the complete control of the participant and may be most susceptible to person‐focused empowerment.

Although DiabetesWise seems to encourage self‐advocacy at the person level, equitable access cannot be achieved without provider‐ and system‐level interventions that address the barriers between device requests and device uptake. One type of inequity observed in the current study was that participants who received diabetes care through a specialty diabetes practice were more likely to receive a prescription and more likely to start a new device than those followed by primary care. Although prior literature has shown access to a certified diabetes care and education specialist (CDCES),^20^ post hoc analyses in the current study did not show a significant relation between access to a CDCES and requesting or receiving prescriptions or new device uptake after interacting with DiabetesWise (all *p* > .05). Several possible barriers outside of CDCES access could explain this pattern. Compared to primary care providers, diabetes practices likely have more comprehensive understanding and familiarity with diabetes devices and greater experience writing and processing prescriptions for such devices. Given that the majority of adults living with diabetes receive their diabetes care through primary care rather than endocrinology,^26^ interventions are needed to combat these types of barriers in access to devices in these settings. A prime example of such an intervention is Project ECHO,^27^, ^28^ which provides specialized diabetes education, consultation, and resources to primary care providers. DiabetesWise can more effectively target primary care to reach more people with insulin‐requiring diabetes through the provider‐focused component. The provider‐facing component of DiabetesWise was developed after the current study for this purpose by offering a nonbiased resource to enhance diabetes device accessibility to providers and the people they serve. It includes information and resources spanning from device education to best practices to support for placing prescriptions. Such component may serve to combat the barriers to device prescription and uptake outside of the person seeking a new device and can pave the way for further patient empowerment. DiabetesWise also aligns with Agarwal and colleagues’^29^ recommendations to address device disparities: (1) more equitable systems of offering technology, (2) more accessible and approachable technology introduction and education, (3) better inclusion of peer and family supports, and (4) assistance with navigating insurance. The current findings can fuel these efforts: effective and equitable device awareness and education is provided via the person‐centered approach of DiabetesWise.

The current study strengths include large sample size, longitudinal study design, and strong retention. Entirely remote study procedures allowed for a broad study sample that included people from geographically diverse regions, with varying income levels, and receiving diabetes care outside of specialty practices. Study limitations include lack of comparison group, relatively homogenous sample based on race or ethnicity, diabetes type, and insurance type, potential sample bias based on online recruitment methods, as well as reliance on self‐report measures of requesting or receiving prescriptions as well as device uptake. Another limitation is that we did not assess for the out‐of‐pocket cost for devices among participants, which likely contributed toward device requests and uptake. Although these limitations prevent any strong causal conclusions from being drawn, the current findings still serve to expand our understanding of diabetes device uptake in general and specifically following interaction with DiabetesWise. Further research on DiabetesWise use activity patterns, potential differences between people with type 1 and type 2 diabetes, feedback from users on if/how DiabetesWise influenced their diabetes management decisions, as well as potential effects of the provider component of DiabetesWise would greatly contribute to this literature. Exploring ways to ensure that people with limited literacy and/or internet access can also benefit from DiabetesWise is another next step that can further promote equity.

## 5 CONCLUSION

Diabetes devices are life‐changing tools. As an unbranded and person‐centered platform, DiabetesWise appears to promote diabetes device awareness and education and to encourage efforts to obtain a diabetes device, especially among those with elevated distress. Nevertheless, further steps must be taken to ensure that every person living with diabetes can make informed and personalized decisions about their management that are reflected in the care and resources that are provided to them.

## CONFLICT OF INTEREST STATEMENT

No potential conflicts of interest.
